# Supplementary material for: A live bacteria enzyme assay for identification of human disease mutations and drug screening
Source: Nat Biomed Eng. 2025 Apr 30;9(9):1547–56. doi: 10.1038/s41551-025-01391-y (PMC12443593; doi:10.1038/s41551-025-01391-y)
Supplement: Supplementary file 1 — Supplementary Notes 1–4, Fig. 1 and Tables 1–4. [file 41551_2025_1391_MOESM1_ESM.pdf]

nature biomedical engineering

Supplementary information https://doi.org/10.1038/s41551-025-01391-y

A live bacteria enzyme assay for identification of human disease mutations and drug screening

In the format provided by the authors and unedited

SUPPLEMENTARY NOTES

Supplementary Note 1. Comparing effects of AG1 on hu-G6PD-Canton and in vitro experiment.

According to the previous experiment, AG1 maximally activated recombinant G6PD-Canton by 1.7-fold[1]. Using LEICA, we reverse-estimated the activity of G6PD-Canton from the measured growth rate of humanized *E. coli* for G6PD-Canton (**Fig. 1f**) when activated by AG1. Fitted linear regression function of log-enzyme activity vs. growth rate is as follows (**Fig. 1f**).

$$GR = 0.0551A + 0.0459\ (Pearson's\ R^2 = 0.840)$$
$$\Leftrightarrow A = 15.235GR - 0.6117,$$

where GR is the growth rate and A is activity of G6PD.

Without AG1 treatment, the growth rate of humanized *E. coli* for G6PD-Canton was 0.0855 h^{-1} (±0.0047, s.d.; n=10). When maximally activated (with 0.3 μM AG1), the growth rate of the same strain was 0.1028 h^{-1} (±0.0026; s.d.; n=10) (data available in **Source Data**). Relative activities are calculated to be 0.6914±0.0714 (0 μM AG1) and 0.9538±0.0403 (0.3 μM AG1). This gives a 1.38-fold increase, consistent with the previous observation (1.7-fold improvement).

Supplementary Note 2. G6PD inhibitors screened.

1. G6PDi-1

A small molecule inhibitor of G6PDi-1 was recently discovered by high-throughput screening[2]. Reported half-maximal inhibitory concentration (IC_{50}) in *in vitro* settings is 0.07 μM. It exhibited inhibitory effect in cell-based experiments: suppresses T cell (mouse CD8+) cytokine production (at concentration of 10-50 μM) and neutrophil (bone-marrow-derived macrophages) oxidative burst at 50 μM[2].

2. Dehydroepiandrosterone (DHEA)

DHEA is a steroid hormone precursor and its G6PD inhibitory activity has been reported previously. recombinant enzymes[3]. However, cell-based assay indicated a limited bioavailability[2] and G6PD inhibition by indirect mechanisms[4].

3. RRx-001

RRx-001 is one of the interesting classes of energetic compounds. Recently, it has been highlighted as a potential radiosensitizer[5]. Although the mechanisms of chemo/radiosensitizing effect have not fully been understood, its inhibitory activity on G6PD may originate primarily through nitric oxide production[6].

4 and 5. Brimonidine (BMN) and proparacaine (PPC)

These two are widely used ophthalmologic drugs. Brimonidine (BMN) is a selective a_2-adrenergic receptor agonist, mainly used to lower intraocular pressure in the clinical treatment of patients with glaucoma[7,8]. Proparacaine (PPC) is a local anesthetic for surface anesthesia in ophthalmology. In the previous study, BMN has an inhibitory effect of G6PD with IC_{50} of 29.93 μM, while PPC has none[6,9].

6. Metamizol (MMZ; dipyrone)

Metamizol is a pain reliever that belongs to pyrazolones. Pyrazolones have been reported to potentially induce severe hemolytic anemia[10]. Effect of metamizole on G6PD was addressed previously, showing an inhibitory effect both *in vitro* and *in vivo,* although IC_{50} was relatively high (17 mM *in vitro*)[11].

7. 6-aminonicotinamide (6AN)

We used 6AN as a positive control of assay. It is a non-selective inhibitor of NADP-dependent enzymes exhibiting antiproliferative effects on prostate cancer cells[12].

Supplementary Note 3. Potential applications of LEICA in personalized medicine research.

As genome sequencing becomes increasingly accessible, precision medicine emerges as an innovative approach to treat patients by accounting for characteristics of individuals. A fundamental aspect of personalized medicine involves understanding a person's genetic makeup to identify medical interventions that are more likely to succeed while minimizing potential side effects.

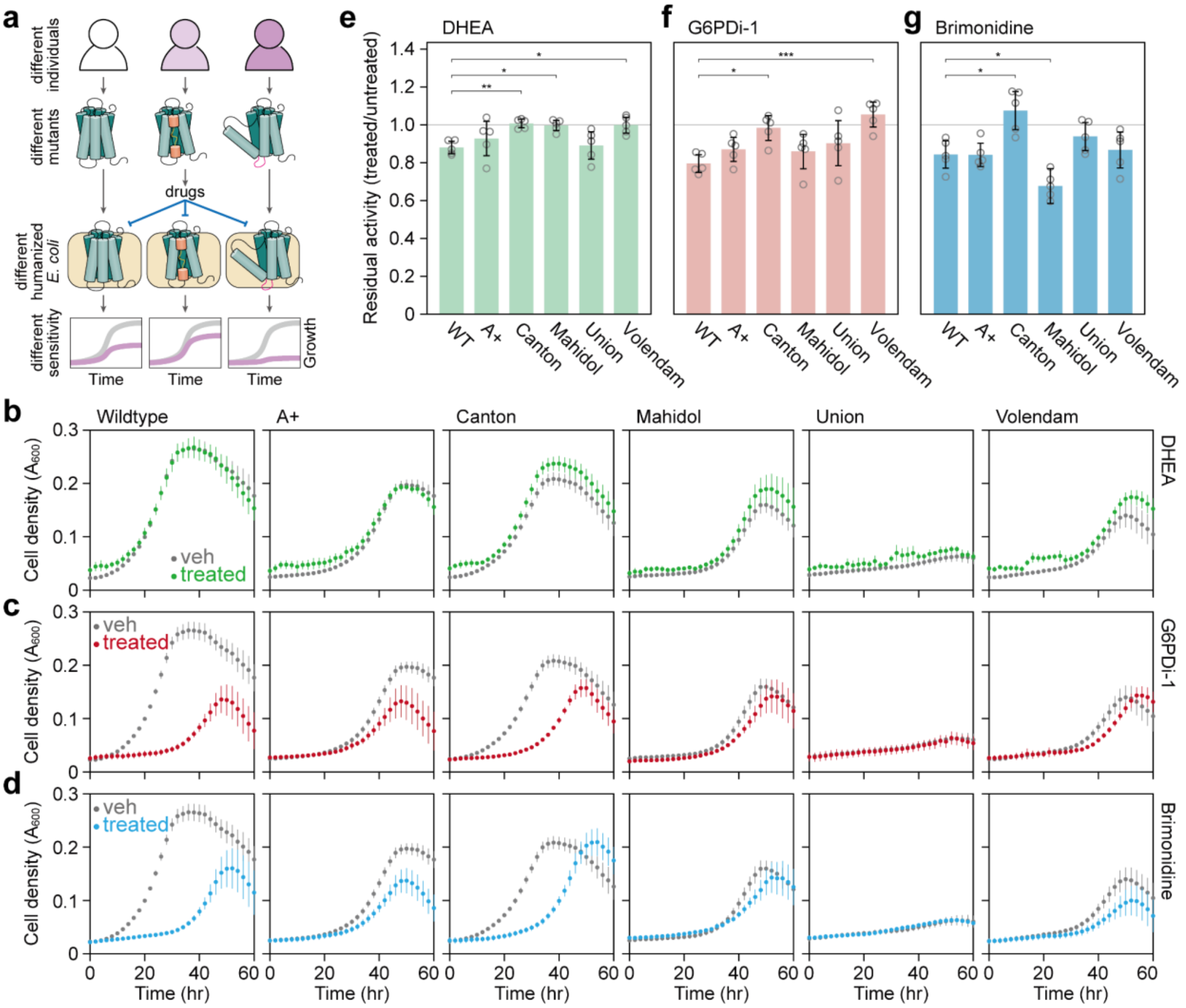

Supplementary Figure 1. Different sensitivities of individual mutants against different compounds. a, LEICA of human G6PD variants screens for different compound sensitivities. **b-d**, Growth profiles of humanized *E. coli* in LEICA with different variants under dehydroepiandrosterone (DHEA) (**b**), inhibitor of G6PD 1 (G6PDi-1) (**c**), and brimonidine (BMN) (**d**) treatments. All compounds were treated with the final concentration of 100 μM. Data are presented as mean values +/− SD. Error bars show SD of five replicates. **e-g,** The growth rates of humanized *E. coli* with treatments compared to the untreated controls represent residual G6PD activities. Variants have different levels of inhibition to DHEA (**e**), G6PDi-1 (**f**), and BMN (**g**). Data are presented as mean values +/− SD. Error bars show SD of five replicates. Dots are individual data points (n=5). **p*-value < 0.05. ***p*-value < 0.005. ****p*-value < 0.001 (two-sided Welch's *t*-test of log-transformed ratios with Bonferroni correction, the residual activities were inferred by comparing treated samples with the untreated samples).

Our proposed assay method has demonstrated the ability to measure the activities of genetic variants and their responses to drug effects, enabling an exploration of each variant's sensitivity to drugs. We subjected DHEA, G6PDi-1, and BMN, which exerted inhibitory effects on the WT G6PD, to all constructed variants to assess differences in sensitivities (**Supplementary Fig. 1a**).

In this assay, variants exhibited different sensitivities to the same treatments (**Supplementary Fig. 1b-d**). Upon DHEA treatment, G6PD-Canton, Mahidol, and Volendam variants showed activities comparable to untreated controls, indicating insensitivities to DHEA (**Supplementary Fig. 1e**). Similarly, G6PD-Canton and Volendam were insensitive to G6PDi-1, while G6PD-A^+, Mahidol, and Union exhibited similar levels of inhibition to the WT (**Supplementary Fig. 1f**). Interestingly, G6PD-Mahidol was more sensitive to BMN than WT G6PD, exhibiting 67.9% residual activity compared to the untreated control, where WT G6PD retained 84.4% activity (**Supplementary Fig. 1g**). In contrast, the G6PD-Canton variant appeared resistant to all three compounds tested (**Supplementary Fig. 1e-g**). These differential drug sensitivities of variants may explain why different G6PD-deficient patients with various G6PD variations exhibit varying levels of hemolytic events after drug administration, such as antimalarials[13]. However, a detailed structural understanding of these drug bindings on G6PD is lacking, necessitating comprehensive molecular investigation into the drug-enzyme interactions.

Supplementary Note 4. Nucleotide sequence of synthesized G6PD.

The following double-stranded DNA fragment was synthesized chemically (Integrated DNA Technologies, USA).

Human G6PD cDNA (1548 bp)

ATGGCAGAGCAGGTGGCCCTGAGCCGGACCCAGGTGTGCGGGATCCTGCGGGAAGAG
CTTTTCCAGGGCGATGCCTTCCATCAGTCGGATACACACATATTCATCATCATGGGTGCA
TCGGGTGACCTGGCCAAGAAGAAGATCTACCCCACCATCTGGTGGCTGTTCCGGGATG
GCCTTCTGCCCGAAAACACCTTCATCGTGGGCTATGCCCGTTCCCGCCTCACAGTGGC
TGACATCCGCAAACAGAGTGAGCCCTTCTTCAAGGCCACCCCAGAGGAGAAGCTCAAG
CTGGAGGACTTCTTTGCCCGCAACTCCTATGTGGCTGGCCAGTACGATGATGCAGCCTC
CTACCAGCGCCTCAACAGCCACATGAATGCCCTCCACCTGGGGTCACAGGCCAACCGC
CTCTTCTACCTGGCCTTGCCCCCGACCGTCTACGAGGCCGTCACCAAGAACATTCACGA
GTCCTGCATGAGCCAGATAGGCTGGAACCGCATCATCGTGGAGAAGCCCTTCGGGAGG
GACCTGCAGAGCTCTGACCGGCTGTCCAACCACATCTCCTCCCTGTTCCGTGAGGACC
AGATCTACCGCATCGACCACTACCTGGGCAAGGAGATGGTGCAGAACCTCATGGTGCT
GAGATTTGCCAACAGGATCTTCGGCCCCATCTGGAACCGGGACAACATCGCCTGCGTTA
TCCTCACCTTCAAGGAGCCCTTTGGCACTGAGGGTCGCGGGGGCTATTTCGATGAATTT
GGGATCATCCGGGACGTGATGCAGAACCACCTACTGCAGATGCTGTGTCTGGTGGCCA
TGGAGAAGCCCGCCTCCACCAACTCAGATGACGTCCGTGATGAGAAGGTCAAGGTGTT
GAAATGCATCTCAGAGGTGCAGGCCAACAATGTGGTCCTGGGCCAGTACGTGGGGAAC
CCCGATGGAGAGGGCGAGGCCACCAAAGGGTACCTGGACGACCCCACGGTGCCCCGC
GGGTCCACCACCGCCACTTTTGCAGCCGTCGTCCTCTATGTGGAGAATGAGAGGTGGG
ATGGGGTGCCCTTCATCCTGCGCTGCGGCAAGGCCCTGAACGAGCGCAAGGCCGAGG
TGAGGCTGCAGTTCCATGATGTGGCCGGCGACATCTTCCACCAGCAGTGCAAGCGCAA
CGAGCTGGTGATCCGCGTGCAGCCCAACGAGGCCGTGTACACCAAGATGATGACCAAG
AAGCCGGGCATGTTCTTCAACCCCGAGGAGTCGGAGCTGGACCTGACCTACGGCAACA
GATACAAGAACGTGAAGCTCCCTGACGCCTATGAGCGCCTCATCCTGGACGTCTTCTGC
GGGAGCCAGATGCACTTCGTGCGCAGCGACGAGCTCCGTGAGGCCTGGCGTATTTTCA
CCCCACTGCTGCACCAGATTGAGCTGGAGAAGCCCAAGCCCATCCCCTATATTTATGGC
AGCCGAGGCCCCACGGAGGCAGACGAGCTGATGAAGAGAGTGGGTTTCCAGTATGAG
GGCACCTACAAGTGGGTGAACCCCCACAAGCTCTAA

SUPPLEMENTARY TABLES

Supplementary Table 1. Human genetic variations in glucose-6-phosphate isomerase (*GPI*) and their clinical manifestations. SNV, single nucleotide variation. P, pathogenic. LP, likely pathogenic. B, benign. LB, likely benign. U, uncertain significance. HA, hemolytic anemia. RBS, red blood cells. ND, not detected. *in vitro* activities of variants were previously reported[14].

Type	Interpretation (#n of evidence in ClinVar; P:LP:B:LB:U)	Nucleotide	AA	Disease association	Historic Name	*in vitro* activity (IU/mg)	Allele frequency (gnomeAD v4.1.0)
-	Normal	-	-	-	Wild-type	400±39.2	-
SNV	benign (0:0:3:3:0)	T623C	**I208T**			NA	0.020571
SNV	likely benign (0:0:0:1:1)	G317A	**R106Q**			NA	0.000229
SNV	pathogenic (2:0:0:0:0)	G1615A	D539N	HA	**Fukuoka**	314±47.1	0.000005
SNV	pathogenic (2:2:0:0:2)	C671T	T224M	HA	**Iwate**	353±45.2	0.000011
SNV	pathogenic (1:0:0:0:0)	C14T	T5I	HA	**Matsumoto**	289±31.7	0.000003
SNV	pathogenic (4:0:0:0:0)	G1040A	**R347H**	HA		NA	0.000017

Supplementary Table 2. Human genetic variations in glucose 6-phosphate dehydrogenase (*G6PD*) and their clinical manifestations. P, pathogenic. LP, likely pathogenic. B, benign. LB, likely benign. U, uncertain significance. HA, hemolytic anemia. AHA, acute hemolytic anemia. CNSHA, chronic non-spherocytic hemolytic anemia. F, favism NA, not available. ND, not detected. Source data are provided as a Source Data file.

Type	Interpretation (#n of evidence in ClinVar; P:LP:B:LB:U)	Nucleotide	AA	Disease association	Historic Name	WHO classification	*in vitro* activity, relative to normal		Allele frequency (gnomeAD v4.1.0)
							k_{cat} (s^{-1})	specific activity (IU/mg)	
SNV	conflicting (9:1:1:3:5)	A376G	N126D	mild HA	**A+**	Class III	0.492±0.003		0.017080
SNV	pathogenic (11:0:0:0:0)	G1376T	R459L	AHA	**Canton**	Class II	0.541±0.010		0.000133
SNV	pathogenic (12:1:0:0:0)	G487A	G163S	AHA	**Mahidol**	Class III	0.818±0.075		0.000038
SNV	pathogenic (15:0:0:0:0)	C1360T	R454C	AHA	**Union**	Class II	0.104±0.005		0.000068
SNV	likely pathogenic (0:1:0:0:0)	C514T	P172S	CNSHA	**Volendam**	Class I	NA	0.171±0.014	ND
SNV	likely pathogenic (0:1:0:0:1)	T196A	F66I	HA	**Songklanagarind**	Class II	0.932±0.028		ND
SNV	pathogenic (1:0:0:0:0)	A713G	K238R	CNSHA	**Durham**	Class I	0.305		ND
SNV	likely pathogenic (0:1:0:0:0)	G488A	G163D	CNSHA	**Plymouth**	Class I	0.992±0.026		ND
SNV	pathogenic (2:2:0:0:0)	T208C	Y70H	HA	**Namoru**	Class II	0.160±0.013		0.000005
SNV	pathogenic (2:3:0:0:0)	A209G	Y70C	HA, F	**Murcia**	Class III	0.903±0.031		0.000006
SNV	pathogenic (1:1:0:0:0)	G593C	R198P	CNSHA	**Santiago**	Class I	0.012±0.001		ND
SNV	likely pathogenic (0:1:0:0:0)	G921C	Q307H	CNSHA	**Omiya**	Class I	0.555±0.013		ND
SNV	pathogenic (2:0:0:0:0)	G1192A	E398K	CNSHA	**Puerto Limon**	Class I	0.652±0.018		ND
SNV	uncertain (0:0:0:0:2)	C1278G	**N426K**						0.000014
SNV	pathogenic (1:0:0:0:0)	T1292G	V431G	HA	**Sumare**				ND
SNV	benign (0:0:1:2:0)	G311A	**R104H**						0.000610
SNV	likely benign (0:0:0:4:3)	G1048C	D350H	HA	**Mira d'Aire**				0.000007

Supplementary Table 3. Human genetic variations in argininosuccinate lyase (*ASL*) and their clinical manifestations. SNV, single nucleotide variation. P, pathogenic. LP, likely pathogenic. B, benign. LB, likely benign. U, uncertain significance. HA, hemolytic anemia. RBS, red blood cells. ND, not detected. a: reference. b: gnomeAD. *in vitro* activities were previously reported[15,16].

Type	Interpretation (#n of evidence in ClinVar; P:LP:B:LB:U)	Nucleotide	AA	Disease association	*in vitro* activity (k_{cat}; s^{-1})	Allele frequency (gnomeAD v4.1.0)
SNV	Normal	-	-	-	34.5±3.0	-
SNV	pathogenic (18:6:0:0:0)	G35A	**R12Q**	ASLD	2.1±0.11	0.001832
SNV	pathogenic (1:2:0:0:0)	A260G	**D87G**	ASLD	0.0	0.000001
SNV	pathogenic (12:0:0:0:0)	A857G	**Q286R**	ASLD	0.6±0.02	0.000091

Supplementary Table 4. Primers used in this study. Overlap extension (OE) PCR was performed to assemble two DNA fragments.

Experiment	Name	Sequence	PCR product (bp)	OE PCR product (bp)
GPI knockout	GPI_KO_SC_F	TTCCAAAGTCACAATTCTCAAAATCAGAAGAGTATTGCTACTAAGGTTGACAATTAATCA	2327	-
	GPI_KO_SC_R	CGGCGTGAACGCCTTATCCGGCCTACATATCGACGATGATACGTGCTCAAGCTTCCTAGG		
zwf knockout	zwf_KO_F	GCGCAAGATCATGTTACCGGTAAAATAACCATAAAGGATAAGCGCAGATAGTGTAGGCTGGAGCTGCTTC	1398	-
	zwf_KO_R	CAAGTATACCCTGGCTTAAGTACCGGGTTAGTTAACTTAAGGAGAATGACGGGATCCGTCGACCTGCAGT		
GPI variants introduction	HsaGPI_F	CTCCAACACCGTTACTTGGGC	1904	2499
	Fukuoka_R	TGGTAGAAGCGTTGTGAGAGGTCACTTGAG		
	Fukuoka_F	GTGACCTCTCACAACGCTTCTACCAATGGG	620	
	HsaGPI_R	CCGCAAGCGCAGATATGGC		
	HsaGPI_F	CTCCAACACCGTTACTTGGGC	958	2499
	Iwate_F	ATGCAGAGATGGCGAAGGAGTGGTTTCTCC		
	Iwate_R	CTCCTTCGCCATCTCTGCATTCGTGATGGT	1561	
	HsaGPI_R	CCGCAAGCGCAGATATGGC		
	HsaGPI_F	CTCCAACACCGTTACTTGGGC	300	2499
	Matsumoto_F	CCGCTCTCATCCGGGACCCCCAGTTCCAGA		
	Matsumoto_R	GGGTCCCGGATGAGAGCGGCCATTAGCAAT	2218	
	HsaGPI_R	CCGCAAGCGCAGATATGGC		
	HsaGPI_F	CTCCAACACCGTTACTTGGGC	603	2499
	R106Q_F	TGCGGAACCAGTCAAACACACCCATCCTGG		
	R106Q_R	GTGTTTGACTGGTTCCGCAGAGCCACGTGC	1915	
	HsaGPI_R	CCGCAAGCGCAGATATGGC		
	HsaGPI_F	CTCCAACACCGTTACTTGGGC	910	2499
	I208T_F	CTGTTCATCACTGCCTCCAAGACCTTTACT		
	I208T_R	CTTGGAGGCAGTGATGAACAGGGAGGACTC	1610	
	HsaGPI_R	CCGCAAGCGCAGATATGGC		
	HsaGPI_F	CTCCAACACCGTTACTTGGGC	1327	2499
	R347H_F	TACCTGCACCACTTTGCTGCGTACTTCCAG		
	R347H_R	CGCAGCAAAGTGGTGCAGGTACTGGTCATA	1193	
	HsaGPI_R	CCGCAAGCGCAGATATGGC		
HsaG6PD and variants cloning	pTrc_bb_F	GTTTAAACGGTCTCCAGCTT	4282	-
	pTrc_bb_R	GGTTTATTCCTCCTTATTTAATCG		
	HsaG6PD_F	TAAATAAGGAGGAATAAACCATGGCAGAGCAGGTGGCCCT	1591	-
	HsaG6PD_R	AAGCTGGAGACCGTTTAAACTTATTAGAGCTTGTGGGGGTTCA		
	HsaG6PD_F	TAAATAAGGAGGAATAAACCATGGCAGAGCAGGTGGCCCT	406	1591
	Aplus_R	TGGAGGGCATCCATGTGGCTGTTGAGGCGC		
	Aplus_F	AGCCACATGGATGCCCTCCACCTGGGGTCA	1205	
	HsaG6PD_R	AAGCTGGAGACCGTTTAAACTTATTAGAGCTTGTGGGGGTTCA		

	HsaG6PD_F	TAAATAAGGAGGAATAAACCATGGCAGAGCAGGTGGCCCT	1405	1591
	Canton_R	CAGGCCTCAAGGAGCTCGTCGCTGCGCACG		
	Canton_F	ACGAGCTCCTTGAGGCCTGGCGTATTTTCA	205	
	HsaG6PD_R	AAGCTGGAGACCGTTTAAACTTATTAGAGCTTGTGGGGGTTCA		
	HsaG6PD_F	TAAATAAGGAGGAATAAACCATGGCAGAGCAGGTGGCCCT	516	1591
	Mahidol_R	GGTTCCAGCTTATCTGGCTCATGCAGGACT		
	Mahidol_F	GAGCCAGATAAGCTGGAACCGCATCATCGT	1095	
	HsaG6PD_R	AAGCTGGAGACCGTTTAAACTTATTAGAGCTTGTGGGGGTTCA		
	HsaG6PD_F	TAAATAAGGAGGAATAAACCATGGCAGAGCAGGTGGCCCT	1389	1591
	Union_R	CGTCGCTGCACACGAAGTGCATCTGGCTCC		
	Union_F	CACTTCGTGTGCAGCGACGAGCTCCGTGAG	221	
	HsaG6PD_R	AAGCTGGAGACCGTTTAAACTTATTAGAGCTTGTGGGGGTTCA		
	HsaG6PD_F	TAAATAAGGAGGAATAAACCATGGCAGAGCAGGTGGCCCT	543	1591
	Volendam_R	TCCCGAAGGACTTCTCCACGATGATGCGGT		
	Volendam_F	CGTGGAGAAGTCCTTCGGGAGGGACCTGCA	1068	
	HsaG6PD_R	AAGCTGGAGACCGTTTAAACTTATTAGAGCTTGTGGGGGTTCA		
	HsaG6PD_F	TAAATAAGGAGGAATAAACCATGGCAGAGCAGGTGGCCCT	225	1591
	Songklanagarind_R	CCACGATGATGGTGTTTTCGGGCAGAAGGC		
	Songklanagarind_F	GAAAACACCATCATCGTGGGCTATGCCCGT	1385	
	HsaG6PD_R	AAGCTGGAGACCGTTTAAACTTATTAGAGCTTGTGGGGGTTCA		
	HsaG6PD_F	TAAATAAGGAGGAATAAACCATGGCAGAGCAGGTGGCCCT	740	1591
	Durham_R	GGGCTCCCTGAAGGTGAGGATAACGCAGGC		
	Durham_F	CTCACCTTCAGGGAGCCCTTTGGCACTGAGG	869	
	HsaG6PD_R	AAGCTGGAGACCGTTTAAACTTATTAGAGCTTGTGGGGGTTCA		
	HsaG6PD_F	TAAATAAGGAGGAATAAACCATGGCAGAGCAGGTGGCCCT	516	1591
	Plymouth_R	GGTTCCAGTCTATCTGGCTCATGCAGGACTCG		
	Plymouth_F	GCCAGATAGACTGGAACCGCATCATCGTGG	1093	
	HsaG6PD_R	AAGCTGGAGACCGTTTAAACTTATTAGAGCTTGTGGGGGTTCA		
	HsaG6PD_F	TAAATAAGGAGGAATAAACCATGGCAGAGCAGGTGGCCCT	236	1591
	Namoru_R	ACGGGCATGGCCCACGATGAAGGTGTTTTC		
	Namoru_F	CATCGTGGGCCATGCCCGTTCCCGCCTCAC	1374	
	HsaG6PD_R	AAGCTGGAGACCGTTTAAACTTATTAGAGCTTGTGGGGGTTCA		
	HsaG6PD_F	TAAATAAGGAGGAATAAACCATGGCAGAGCAGGTGGCCCT	236	1591
	Murcia_R	ACGGGCACAGCCCACGATGAAGGTGTTTTC		
	Murcia_F	ATCGTGGGCTGTGCCCGTTCCCGCCTCACA	1373	
	HsaG6PD_R	AAGCTGGAGACCGTTTAAACTTATTAGAGCTTGTGGGGGTTCA		
	HsaG6PD_F	TAAATAAGGAGGAATAAACCATGGCAGAGCAGGTGGCCCT	621	1591
	Santiago_R	GGTCGATGGGGTAGATCTGGTCCTCACGGAAC		
	Santiago_F	AGATCTACCCCATCGACCACTACCTGGGCA	988	
	HsaG6PD_R	AAGCTGGAGACCGTTTAAACTTATTAGAGCTTGTGGGGGTTCA		
	HsaG6PD_F	TAAATAAGGAGGAATAAACCATGGCAGAGCAGGTGGCCCT	948	1591

	Omiya_R	CCACGTAGTGGCCCAGGACCACATTGTTGG		
	Omiya_F	TCCTGGGCCACTACGTGGGGAACCCCGATG	661	
	HsaG6PD_R	AAGCTGGAGACCGTTTAAACTTATTAGAGCTTGTGGGGGTTCA		
	HsaG6PD_F	TAAATAAGGAGGAATAAACCATGGCAGAGCAGGTGGCCCT	1222	1591
	Puerto Limon_R	TACACGGCCTTGTTGGGCTGCACGCGGATC		
	Puerto Limon_F	CAGCCCAACAAGGCCGTGTACACCAAGATG	389	
	HsaG6PD_R	AAGCTGGAGACCGTTTAAACTTATTAGAGCTTGTGGGGGTTCA		
	HsaG6PD_F	TAAATAAGGAGGAATAAACCATGGCAGAGCAGGTGGCCCT	340	1591
	R104H_R	TAGGAGTTGTGGGCAAAGAAGTCCTCCAGC		
	R104H_F	TCTTTGCCCACAACTCCTATGTGGCTGGCC	1270	
	HsaG6PD_R	AAGCTGGAGACCGTTTAAACTTATTAGAGCTTGTGGGGGTTCA		
	HsaG6PD_F	TAAATAAGGAGGAATAAACCATGGCAGAGCAGGTGGCCCT	1077	1591
	Mira d'Aire_R	GCACCCCATGCCACCTCTCATTCTCCACATAGAGG		
	Mira d'Aire_F	GAGAGGTGGCATGGGGTGCCCTTCATCCTG	533	
	HsaG6PD_R	AAGCTGGAGACCGTTTAAACTTATTAGAGCTTGTGGGGGTTCA		
	HsaG6PD_F	TAAATAAGGAGGAATAAACCATGGCAGAGCAGGTGGCCCT	1307	1591
	N426K_R	CTTGTATCTCTTGCCGTAGGTCAGGTCCAG		
	N426K_F	CTACGGCAAGAGATACAAGAACGTGAAGCTCCC	303	
	HsaG6PD_R	AAGCTGGAGACCGTTTAAACTTATTAGAGCTTGTGGGGGTTCA		
	HsaG6PD_F	TAAATAAGGAGGAATAAACCATGGCAGAGCAGGTGGCCCT	1321	1591
	Sumare_R	GGGAGCTTCCCGTTCTTGTATCTGTTGCCGT		
	Sumare_F	ACAAGAACGGGAAGCTCCCTGACGCCTATG	289	
	HsaG6PD_R	AAGCTGGAGACCGTTTAAACTTATTAGAGCTTGTGGGGGTTCA		
argH knockout	argH_KO_F	CCGGAGGCGCAGCTTTCCGGCATTGAATTTCAAAATAAGGAAACAGAGTTGTGTAGGCTGGAGCTGCTTC	1394	-
	argH_KO_R	TAAAAAAGCCCGGCGATAAGCCAGGCTCAAATTTATACATATAAATGTTCTCCGTCGACCTGCAGTTCGA		
HsaASL and variants cloning	HsaASL_F	TAAATAAGGAGGAATAAACCATGGCCTCGGAGAGTGGGAA	1438	-
	HsaASL_R	AAGCTGGAGACCGTTTAAACTTATTAGGCCTGCTGTGCCTGCAGTA		
	R12Q_F	GGGAAGCTTTGGGGTGGCCAGTTTGTGGGTGCAGTGGACC	1403	-
	HsaASL_R	AAGCTGGAGACCGTTTAAACTTATTAGGCCTGCTGTGCCTGCAGTA		
	R12Q_F_primer extension	TAAATAAGGAGGAATAAACCATGGCCTCGGAGAGTGGGAAGCTTTGGGGTGGCCA	1438	
	HsaASL_R	AAGCTGGAGACCGTTTAAACTTATTAGGCCTGCTGTGCCTGCAGTA		
	HsaASL_F	TAAATAAGGAGGAATAAACCATGGCCTCGGAGAGTGGGAA	289	1438
	D87G_R	GTGTGGATGCCCTCATCATTGGAGTTCAGTTTG		
	D87G_F	ATGATGAGGGCATCCACACAGCCAATGAGC	1168	
	HsaASL_R	AAGCTGGAGACCGTTTAAACTTATTAGGCCTGCTGTGCCTGCAGTA		
	HsaASL_F	TAAATAAGGAGGAATAAACCATGGCCTCGGAGAGTGGGAA	887	1438
	Q286R_R	GTTTTTCTTCCGGGGCATCAGGCTGCTTCC		
	Q286R_F	TGATGCCCCGGAAGAAAAACCCCGACAGTTTGG	571	
	HsaASL_R	AAGCTGGAGACCGTTTAAACTTATTAGGCCTGCTGTGCCTGCAGTA		

SUPPLEMENTARY REFERENCES

1. Hwang, S. *et al.* Correcting glucose-6-phosphate dehydrogenase deficiency with a small-molecule activator. *Nat. Commun.* **9**, 4045 (2018).
2. Ghergurovich, J. M. *et al.* A small molecule G6PD inhibitor reveals immune dependence on pentose phosphate pathway. *Nat. Chem. Biol.* **16**, 731–739 (2020).
3. Marks, P. A. & Banks, J. INHIBITION OF MAMMALIAN GLUCOSE-6-PHOSPHATE DEHYDROGENASE BY STEROIDS. *Proc. Natl. Acad. Sci. U. S. A.* **46**, 447–452 (1960).
4. Ho, H.-Y., Cheng, M.-L., Chiu, H.-Y., Weng, S.-F. & Chiu, D. T.-Y. Dehydroepiandrosterone induces growth arrest of hepatoma cells via alteration of mitochondrial gene expression and function. *Int. J. Oncol.* **33**, 969–977 (2008).
5. Oronsky, B. *et al.* RRx-001, a novel clinical-stage chemosensitizer, radiosensitizer, and immunosensitizer, inhibits glucose 6-phosphate dehydrogenase in human tumor cells. *Discov. Med.* **21**, 251–265 (2016).
6. Yalcin, O. *et al.* From METS to malaria: RRx-001, a multi-faceted anticancer agent with activity in cerebral malaria. *Malar. J.* **14**, 218 (2015).
7. Quigley, H. A. & Broman, A. T. The number of people with glaucoma worldwide in 2010 and 2020. *Br. J. Ophthalmol.* **90**, 262–267 (2006).
8. Zhou, X., Zhang, T. & Wu, J. Brimonidine enhances inhibitory postsynaptic activity of OFF- and ON-type retinal ganglion cells in a Wistar rat chronic glaucoma model. *Exp. Eye Res.* **189**, 107833 (2019).
9. Çalışkan, B., Öztürk Kesebir, A., Demir, Y. & Akyol Salman, İ. The effect of brimonidine and proparacaine on metabolic enzymes: Glucose-6-phosphate dehydrogenase, 6-phosphogluconate dehydrogenase, and glutathione reductase. *Biotechnol. Appl. Biochem.* **69**, 281–288 (2022).
10. Sansone, G., Reali, S., Sansone, R. & Allegranza, F. Acute hemolytic anemia induced by a pyrazolonic drug in a child with glucose-6-phosphate dehydrogenase deficiency. *Acta Haematol.* **72**, 285–287 (1984).
11. Ciftçi, M., Ozmen, I., Büyükokuroğlu, M. E., Pençe, S. & Küfrevioğlu, O. I. Effects of metamizol and magnesium sulfate on enzyme activity of glucose 6-phosphate dehydrogenase from human erythrocyte *in vitro* and rat erythrocyte *in vivo*. *Clin. Biochem.* **34**, 297–302 (2001).
12. Tsouko, E. *et al.* Regulation of the pentose phosphate pathway by an androgen receptor–mTOR-mediated mechanism and its role in prostate cancer cell growth. *Oncogenesis* **3**, e103–e103 (2014).
13. Bancone, G. & Chu, C. S. G6PD Variants and Haemolytic Sensitivity to Primaquine and Other Drugs. *Front. Pharmacol.* **12**, 638885 (2021).
14. Kanno, H., Fujii, H. & Miwa, S. Expression and enzymatic characterization of human

glucose phosphate isomerase (GPI) variants accounting for GPI deficiency. *Blood Cells Mol Dis* **24**, 54–61 (1998).

15. Sampaleanu, L. M., Vallée, F., Thompson, G. D. & Howell, P. L. Three-dimensional structure of the argininosuccinate lyase frequently complementing allele Q286R. *Biochemistry* **40**, 15570–15580 (2001).
16. Yu, B., Thompson, G. D., Yip, P., Howell, P. L. & Davidson, A. R. Mechanisms for intragenic complementation at the human argininosuccinate lyase locus. *Biochemistry* **40**, 15581–15590 (2001).
